# Fairness evaluation of collaborative governance of food safety in Jilin Province based on AHP-FCE model: multi-dimensional difference analysis and policy implications research

**DOI:** 10.3389/fpubh.2026.1770663

**Published:** 2026-03-03

**Authors:** Xiaodan Qi, Yuxin Chen, Xihe Yu

**Affiliations:** School of Public Health, Jilin University, Changchun, Jilin, China

**Keywords:** AHP-FCE framework, DPSIR model, food safety, government regulation, policy

## Abstract

The distribution of food safety risks and their associated health impacts constitute a significant societal cause of health inequalities. As a major agricultural and food production base in China, Jilin Province has seen limited research on the fairness of collaborative food safety governance from a health equity perspective, despite its critical necessity. This study integrates the Analytic Hierarchy Process (AHP) with Fuzzy Comprehensive Evaluation (FCE) to establish an evaluation framework. Following a “structure-process-outcome” analytical approach and the DPSIR model, a multidimensional indicator system was constructed. Comprehensive data from multiple sources—including policy documents, regulatory enforcement records, sampling inspection data, and questionnaire surveys—were synthesized with expert consultation results from 33 specialists to conduct a quantitative assessment of the fairness in Jilin Province’s collaborative food safety governance. Results indicate that the comprehensive score for local collaborative food safety governance is 3.63/5, reflecting an overall upper-middle level. However, significant urban–rural and socio-economic disparities in governance fairness were identified. Key findings include: (1) Distinct urban–rural and regional gradients in governance resource allocation (e.g., regulatory density, testing coverage) and significant differences in public information awareness, participation convenience, and satisfaction. (2) Multi-dimensional performance is uneven—government regulatory capacity scored highest (4.0), corporate responsibility implementation lowest (3.0), while third-party testing and public participation both scored 3.5, indicating insufficient depth of social force involvement. (3) Structural equity and process equity are key determinants of outcome equity, with groups possessing higher socioeconomic capital demonstrating stronger capacity to benefit from the governance system. (4) The overall coordination of the governance system remains inadequate, exhibiting characteristics of “strong government, weak society and enterprises,” with insufficient information sharing and collaboration mechanisms. The study indicates that Jilin Province’s food safety governance exhibits partial inequities in benefit distribution. Based on these findings, this paper proposes corresponding policy implications to serve as a reference for improving food management and alleviating related challenges.

## 1 Introduction

With rapid socioeconomic development and industrial restructuring, the food supply chain continues to expand, making food safety issues increasingly complex and systemic. This poses a significant threat to public health, local economies, and government credibility worldwide (1). The growing interconnectedness of the global food market further exacerbates this complexity, demanding robust food safety management systems across diverse geopolitical landscapes (2). In food safety incidents, a substantial proportion of foodborne illnesses originate from household-consumed foods (3), highlighting the broad impact of food safety across the entire consumption chain. In China, despite notable progress in food safety governance, the development of food safety frameworks remains uneven and inadequate due to inherent regional, urban–rural, and dual institutional structures, presenting numerous challenges (4). Food safety incidents not only affect public health but also exert complex and heterogeneous impacts on food prices and regional economies. According to the State Administration for Market Regulation’s inspection report, China completed 6.7949 million batches of food safety supervision sampling in 2024, with an overall non-compliance rate of 2.96% (5). Among these, the Administration conducted 16,814 batches of inspections at its own level, reporting 244 non-compliant batches—a 141.58% increase from the 101 non-compliant batches in 2023. Lower non-compliance rates were observed in high-consumption food categories, including grain products (0.54%), edible oils (0.72%), meat products (0.81%), egg products (0.12%), and dairy products (0.09%). Key issues identified included pesticide residue exceeding standards (39.56%) and microbial contamination (16.73%), with some fruit products showing excessive total bacterial counts; 85 batches exhibited improper food additive usage, such as illegal benzoic acid and its sodium salts in dried wood ear mushrooms, and excessive cyclamate in flower rolls (6). Additionally, 66 batches failed quality standards, including walnut oil with excessive peroxide values and special dietary foods with non-compliant nutritional content. Frequent high-profile safety incidents occurred (7), including three Anhui pre-cooked meal manufacturers exposed for using “offcuts” containing excessive lymph nodes and lipomas to produce preserved vegetable pork belly, and “Tinghua wine” falsely advertising medical benefits like “boosting immunity” and “anti-aging.”

Food safety risk management is recognized as a critical cross-sectoral issue requiring interagency collaboration. However, implementation often suffers from low coordination levels, inadequate process oversight, limited information sharing, and incomplete disclosure (8, 9). China’s current food safety governance system frequently faces challenges such as overlapping departmental responsibilities, insufficient implementation of corporate accountability, and limited public participation, urgently necessitating enhanced collaborative governance efficiency. As a major agricultural and food processing hub, Jilin Province’s food safety governance effectiveness directly impacts regional livelihood security and industrial high-quality development.

The Global Food Safety Initiative (GFSI) has recognized the importance of adopting an interdisciplinary approach to detect and reduce food fraud. Within global food safety management practices, the Hazard Analysis and Critical Control Points (HACCP) system is widely regarded as an effective tool for preventing foodborne illnesses. It assists businesses in establishing a scientific and systematic food safety management system, thereby ensuring product safety and compliance. HACCP serves as a core component of numerous national and international standards (10). As a systematic preventive approach, HACCP meticulously analyzes each stage of the food production process to identify potential physical, chemical, and biological hazards. It establishes Critical Control Points (CCPs) to prevent, eliminate, or reduce these hazards to acceptable levels, thereby identifying, evaluating, and controlling significant threats to food safety. Current academic research on food safety governance primarily focuses on regulatory policies, risk prevention and control, and traceability system development (11, 12). Studies by Hafiz Muhammad Rizwan Abid (11) and others indicate that internet-based tools are increasingly popular for tracking, tracing, and ensuring food safety throughout the food chain, underscoring the necessity of integrated systems across the food supply chain. Yi et al. (4) further discuss how institutional pressures influence food safety governance within emerging supply chains. By examining the complex interplay between food safety incidents, public health concerns, and food price risks—along with the dynamic and uncertain nature of risk transmission—they construct an integrated conceptual framework encompassing institutional pressures, food safety practices, and governance effectiveness.

However, existing research exhibits significant limitations in quantitatively assessing the performance of multi-stakeholder collaborative governance, particularly regarding the inherent ambiguity and hierarchical nature of complex food safety systems. The suitability of relevant evaluation frameworks remains inadequately addressed. Traditional quantitative studies often rely on single evaluation indicators or expert subjective judgments, lacking systematic consideration of the multidimensional and cross-level attributes of food safety governance performance. This approach may struggle to comprehensively and accurately capture the core essence and actual level of food safety performance. As demonstrated in Yingxue Ren’s research (13), the core control mechanisms and key safeguards underpinning food safety performance enhancement exhibit multidimensional complexity. This further underscores the academic value and practical necessity of developing a comprehensive evaluation methodology that integrates systematicity, comprehensiveness, and adaptability.

This study, grounded in the provincial context of Jilin, aims to construct a scientific indicator system for collaborative food safety governance. It comprehensively captures the multifaceted characteristics of food safety management, proposes optimization recommendations for collaborative governance tailored to Jilin Province, and provides decision support for local governments to refine their food safety governance systems. This enhances understanding and practice in food safety governance. The DPSIR model originated from the need for comprehensive assessment of complex environmental issues. a structured tool promoted by the European Environment Agency (EEA) to describe environmental issues and their management responses. It views environmental systems as dynamic processes whose health is shaped by five interconnected elements: drivers, pressures, state, impacts, and responses. Applied to food safety management, it facilitates more systematic analysis of the causes, manifestations, consequences, and countermeasures of food safety issues, helping policymakers and researchers understand the relationship between environmental changes and human activities (14). To optimize the evaluation of food safety management systems, this study constructed a performance evaluation system for collaborative governance of food safety in Jilin Province based on the DPSIR framework. Quantitative analysis was conducted using the Analytic Hierarchy Process (AHP) and Fuzzy Comprehensive Evaluation (FCE) methods. This integrated AHP-FCE framework systematically deconstructs complex multi-criteria decision problems into hierarchical structural models while effectively addressing inherent fuzziness and uncertainty in human subjective judgments and qualitative factors. Through empirical research methods including field interviews, expert consultations, and structured scoring, this study identifies and selects key indicators influencing collaborative governance performance, subsequently employing fuzzy comprehensive evaluation to quantify governance levels.

This research demonstrates significant innovative value at both theoretical and practical levels. Theoretically, by integrating the systemic thinking of collaborative governance theory, the deconstruction logic of the Analytic Hierarchy Process (AHP), and the quantitative capabilities of fuzzy set theory for uncertainty, this study constructs an integrated evaluation framework. This not only effectively fills a research gap in multi-stakeholder collaborative performance quantification methodologies but also advances the instrumentalization and empirical development of public management theory within complex Chinese contexts. It provides new analytical perspectives and theoretical tools for deciphering the interactive efficacy of diverse governance actors. Practically, the developed framework overcomes the limitations of traditional evaluations—which are dominated by qualitative approaches and static, singular perspectives—by enabling precise diagnosis and dynamic assessment of regional food safety collaborative governance levels through quantifiable, structured, and model-based pathways. The research outcomes provide regulatory agencies with decision support featuring clear operational guidance, aiding in optimizing governance resource allocation and refining collaborative mechanism design. This ultimately enhances the overall effectiveness and resilience of the food safety governance system.

## 2 Materials and methods

### 2.1 Design of index system

#### 2.1.1 Theoretical framework

To scientifically assess the level of collaborative governance of food safety in Jilin Province, based on the Driving Force-Pressure-State-Impact-Response (DPSIR) framework, and in combination with the practical characteristics of food safety governance and the requirements of collaborative management, this paper constructs a multi-level and multi-dimensional evaluation index system. This system adheres to the principles of scientificity, systematicness, comparability, and operability. It fully absorbs research achievements in the fields of collaborative governance (15, 16), public management performance and food safety risk assessment both at home and abroad, and combines the actual governance practice in Jilin Province to form four first-level indicators centered on government regulatory capacity, enterprise responsibility implementation, third-party and testing quality, and public participation.

Each first-level indicator is further refined into several second-level indicators, aiming to comprehensively reflect the operational status and collaborative effectiveness of the food safety governance system in Jilin Province. For instance, the government’s regulatory capacity mainly examines the coverage rate of regulatory personnel, law enforcement norms, departmental coordination, and the soundness of regulatory systems. The implementation of corporate responsibility emphasizes the self-examination and rectification capabilities as well as the traceability rate. The third party and testing quality include the blind sample pass rate and the rate of third-party violations being investigated and dealt with. Public participation is reflected in the popularization of safety knowledge, public satisfaction, and the degree of participation in public consultation, etc.

Through this multi-dimensional decomposition, the indicator system can systematically reflect the structural characteristics and dynamic evolution laws of the collaborative governance of food safety, providing a solid foundation for subsequent quantitative analysis. On this basis, a comprehensive model combining the Analytic Hierarchy Process (AHP) and the Fuzzy Comprehensive Evaluation Method (FCE) is introduced. AHP is used to determine the relative weights of each element in the index system, while FCE is used to handle the fuzziness and subjectivity in expert scoring, achieving a quantitative comprehensive evaluation (17–20).

**Figure 1:**
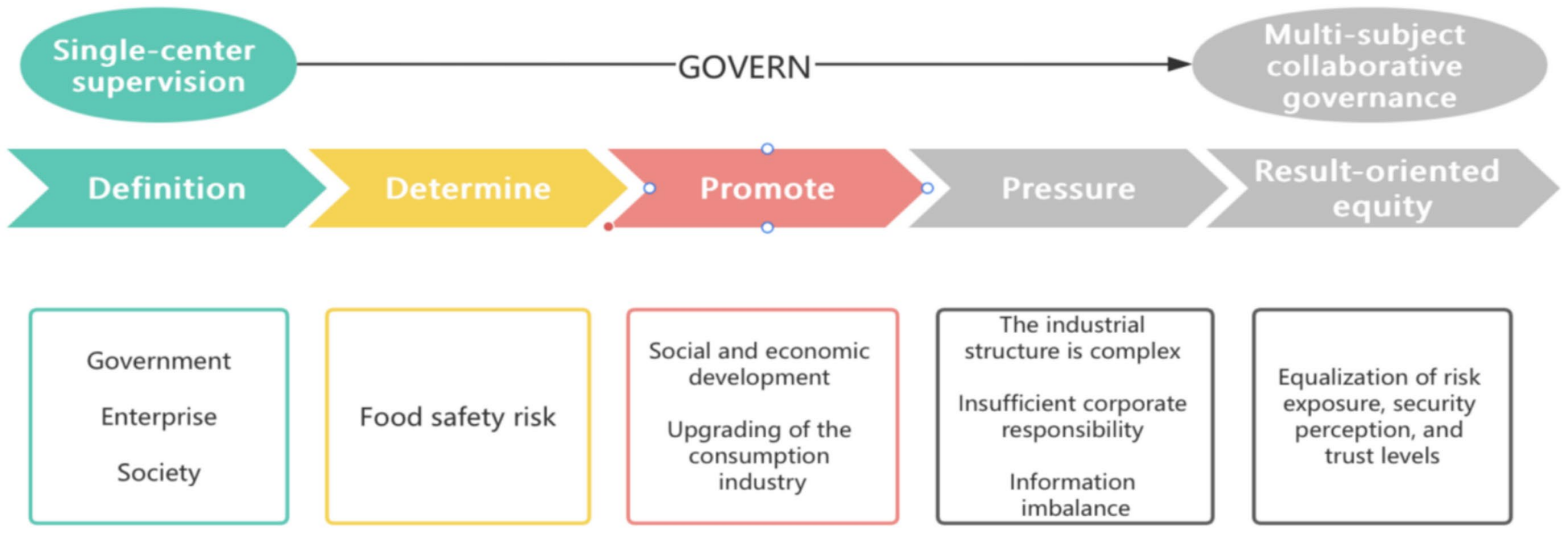
The operation of the collaborative governance system for food safety based on DPSIR.

The hierarchical structure of the AHP model for this study is depicted in Figure 1. The top level (Goal) represents the comprehensive evaluation of fairness in collaborative food safety governance. The second level (Criteria) consists of the four first-level indicators: Government Regulatory Capacity (A1), Implementation of Corporate Responsibility (A2), Third Parties and Inspection Quality (A3), and Public Participation (A4). The third level (Sub-criteria) comprises the 18 specific evaluation indicators (G1–G5, E1–E5, T1–T4, C1–C4) as detailed in Table 1. This visual representation clarifies the prioritization logic and the relationship between the overall objective, evaluation dimensions, and specific measurement points.

**Table 1:** The experts’ characteristics, answered AHP questionnaire.

Person	Workplace	Education level	Work experience (year)	Sex
P1	GRA	PhD	15	M
P2	FPE	MMS	18	M
P3	TTI	MA	19	F
P4	FPE	PhD	11	F
P5	GRA	MA	14	F
P6	TTI	MA	11	M
P7	GRA	BA	8	F
P8	FPE	MA	15	M
P9	CO	PhD	17	M
P10	TTI	BA	5	F
P11	GRA	MS	16	F
P12	FPE	MMS	11	F
P13	CO	MS	9	M
P14	GRA	BA	14	M
P15	CO	MS	13	F
P16	TTI	MA	10	M
P17	GRA	PhD	9	M
P18	FPE	MS	6	F
P19	GRA	MA	11	F
P20	CO	BA	13	F
P21	CO	MMS	12	M
P22	FPE	MS	5	M
P23	GRA	MS	9	M
P24	TTI	BA	7	F
P25	CO	MMS	17	F
P26	FPE	MA	15	F
P27	CO	MA	5	F
P28	GRA	MS	14	F
P29	CO	PhD	18	M
P30	GRA	MA	11	M
P31	TTI	MS	14	F
P32	GRA	MMS	13	F
P33	GRA	PhD	7	F

GRA, Government Regulatory Agencies; FPE, Food Production Enterprises; TTI, Third-party Testing Institutions; CO, Consumer Organizations. MMS, Master of Medical Science; MA, Master of Art; MS, Master of Science; BA, Bachelor of Art; M, Male; F, Female.

#### 2.1.2 Data sources and sample construction

This study adopts a combination of expert scoring and semi-structured interviews to replace the traditional pure statistical data method, in order to fully reflect governance synergy and multi-subject perspectives. From May to September 2025, the research team conducted on-site investigations in Changchun, Tonghua, Baishan, Yanbian and other places in Jilin Province. The interviewees included government regulatory agencies, food production enterprises, third-party testing institutions and consumer organizations, with a total of 33 respondents, and all participants gave informed consent to this study. The characteristic of 33 experts is presented in Table 1.

The specific sample composition is as follows: 15 heads of government regulatory departments (including market supervision, agriculture and rural affairs, health and wellness, education, etc.); there are 8 people in the management team of a food enterprise. Six experts from testing and certification institutions; four representatives from consumer associations and social organizations.

Experts score the importance and implementation of the secondary and tertiary indicators of the collaborative governance of food safety based on practical experience, financial data, statistical yearbooks and case files of previous years. The scoring was conducted using a five-level Likert scale (1–5 points), and a judgment matrix was formed according to the painwise comparison method, providing basic data for the calculation of AHP weights.

#### 2.1.3 Establishment of index system

Based on the results of previous literature research, policy analysis and interviews, a performance evaluation index system for collaborative governance of food safety in Jilin Province was constructed, which includes 4 secondary indicators and 18 tertiary indicators, covering four dimensions: government regulatory capacity, enterprise responsibility implementation, third-party and testing quality, and public participation, showing in Table 2.

**Table 2:** Selection of evaluation indicators for collaborative governance of food safety.

First-level indicator	Second-level indicators	Third-level indicators
A. The effect of collaborative governance on food safety	A1. Government regulatory capacity (G)	Supervise the Gini coefficient/urban–rural ratio of human resources and funds (G1)
		The coverage and targeting of food safety supervision and sampling inspection (G2)
		The timeliness of responses to illegal acts and regional differences (G3)
		The frequency and case type distribution of cross-departmental collaborative law enforcement (G4)
		The transparency and understandability of regulatory information disclosure (G5)
	A2. Implementation of corporate responsibility (E)	The certification rates of food safety management systems in enterprises of different scales (E1)
		The enterprise scale gradient of traceability information completeness (E2)
		The differences in the coverage and effectiveness of training for practitioners (E3)
		The authenticity of the self-inspection report submission rate and the problem discovery rate (E4)
		Industry and scale differences in the proportion of food safety investment to revenue (E5)
	A3. Third parties and inspection quality (T)	The regional equilibrium index of the distribution of testing institution outlets (T1)
		The transparency of service charge standards and the affordability of small and micro enterprises (T2)
		The stability of the timeliness of issuing test reports and the fulfillment rate of commitments (T3)
		The openness of proficiency testing and flight inspection results and the differences among institutions (T4)
	A4. Public participation (C)	Group differences in food safety risk perception and knowledge level (C1)
		Analysis of the Differences in the Utilization Efficiency of Complaint and Reporting Channels (C2)
		The representativeness and accessibility of participation in policy consultation and risk communication activities (C3)
		The deviation in the attention paid to food safety issues by the media and social platforms (C4)

### 2.2 Research data processing

#### 2.2.1 Establish a comprehensive evaluation factor set U

The factor set is a collection of elements, where elements represent various factors that affect the object under evaluation. This element set is denoted by U, where U={U1,U2,…,Ui} , where element U_i_ represents the i-th factor that affects the object under evaluation. The evaluation index system consists of 1 first-level indicator, 4 second-level indicators, and 18 third-level indicators. The first-level evaluation index set U = {the effect of collaborative governance of food safety}, the second-level evaluation index set U = {government regulatory capacity U_1_, enterprise responsibility implementation U_2_, public participation U_3_, third-party and testing quality U_4_}, and the third-level evaluation index set U = {Supervise the Gini coefficient/urban–rural ratio of human resources and funds U_1_, The coverage and targeting of food safety supervision and sampling inspection U_2_. The deviation in the attention paid to food safety issues by the media and social platforms U_18_}.

#### 2.2.2 The entropy weight method is used to determine the index weights

First, de-dimensionalize each indicator. Corresponding to the third-level indicator { $X_{1},X_{2},\ldots X_{n}$ }, where $X_{i}$ ={ $x_{1},x_{2},\ldots x_{n}$ }. Assuming that the standardized values of each indicator data are { $Y_{1},Y_{2},\ldots Y_{n}$ }, when the indicator direction is positive, then:


$$Y_{\mathrm{ij}}=\frac{X_{\mathrm{ij}}-\min(X_{\mathrm{ij}})}{\max(X_{i})-\min(X_{i})}$$

The second step is to calculate the ratio of each indicator, that is, the proportion of the j-th indicator in the i-th plan.


$$P_{\mathrm{ij}}=\frac{Y_{\mathrm{ij}}}{\sum_{i=1}^{n}Y_{\mathrm{ij}}},i,j=1,2,3.\ldots ,m$$

The third step is to calculate the information entropy of each indicator. The information entropy of a set of data is:


$$E_{j}=-\ln{\left(n\right)}^{-1}\sum_{i=1}^{n}p_{\mathrm{ij}}\ln p_{\mathrm{ij}}$$

The fourth step is to determine the weights of each indicator. According to the calculation formula of information entropy, the information entropy of each indicator is calculated as E_1_, E_2_,. E_m_. First, calculate the weights of each indicator through information entropy:


$$W_{j}=\frac{1-E_{j}}{k-\sum E_{j}}(j=1,2,\ldots m)$$

Here, k refers to the number of indicators, that is, k = m. The results of the weight calculation are shown in Table 3.

**Table 3:** The weight of food safety collaborative governance indicators.

Second-level indicators	Weight	Third-level indicators	Weight
A1. Government regulatory capacity (G)	0.3850984	Supervise the Gini coefficient/urban–rural ratio of human resources and funds (G1)	0.079366
		The coverage and targeting of food safety supervision and sampling inspection (G2)	0.031488
		The timeliness of responses to illegal acts and regional differences (G3)	0.040207
		The frequency and case type distribution of cross-departmental collaborative law enforcement (G4)	0.034251
		The transparency and understandability of regulatory information disclosure (G5)	0.199787
A2. Implementation of corporate responsibility (E)	0.11727	The certification rates of food safety management systems in enterprises of different scales (E1)	0.022291
		The enterprise scale gradient of traceability information completeness (E2)	0.020426
		The differences in the coverage and effectiveness of training for practitioners (E3)	0.024855
		The authenticity of the self-inspection report submission rate and the problem discovery rate (E4)	0.025572
		Industry and scale differences in the proportion of food safety investment to revenue (E5)	0.024126
A3. Third parties and inspection quality (T)	0.2438468	The regional equilibrium index of the distribution of testing institution outlets (T1)	0.026714
		The transparency of service charge standards and the affordability of small and micro enterprises (T2)	0.104797
		The stability of the timeliness of issuing test reports and the fulfillment rate of commitments (T3)	0.051819
		The openness of proficiency testing and flight inspection results and the differences among institutions (T4)	0.060518
A4. Public participation (C)	0.2537846	Group differences in food safety risk perception and knowledge level (C1)	0.063173
		Analysis of the Differences in the Utilization Efficiency of Complaint and Reporting Channels (C2)	0.038505
		The representativeness and accessibility of participation in policy consultation and risk communication activities (C3)	0.098182
		The deviation in the attention paid to food safety issues by the media and social platforms (C4)	0.053924

#### 2.2.3 Establish a comprehensive evaluation set V

The evaluation set is a collection of results, composed of various possible outcomes that may occur after the evaluator evaluates the evaluated object using evaluation elements. This set is represented by V, V= {V1,V2,…,Vj} , where element V_j_ represents the j-th evaluation result. The elements of the evaluation set can be either qualitative evaluations or quantitative score evaluations. In this paper, the corresponding evaluation set is established as V = {very important, important, average, not important, very not important}, and is assigned values of 5, 4, 3, 2, and 1, respectively.

#### 2.2.4 Determine the fuzzy evaluation matrix R

Based on the expert’s fuzzy scoring, the performance level of each secondary indicator is classified into five grades: excellent (1.0–0.8), good (0.8–0.6), medium (0.6–0.4), average (0.4–0.2), and poor (0.2–0).

By statistically analyzing expert opinions, the fuzzy membership degree matrix R corresponding to each indicator is formed. Some of the results are shown in Table 4.

**Table 4:** Fuzzy membership degree matrix of the second-level indicator G1 (Government regulatory Capacity).

Second-level indicators	Third-level indicators	Very unimportant	Unimportant	Generally	Important	Very important
A1. Government regulatory capacity (G)	Supervise the Gini coefficient/urban–rural ratio of human resources and funds (G1)	0	0	0.294118	0.647059	0.058824
	The coverage and targeting of food safety supervision and sampling inspection (G2)	0	0.058824	0.470588	0.352941	0.117647
	The timeliness of responses to illegal acts and regional differences (G3)	0	0.117647	0.352941	0.470588	0.058824
	The frequency and case type distribution of cross-departmental collaborative law enforcement (G4)	0	0.117647	0.235294	0.647059	0
	The transparency and understandability of regulatory information disclosure (G5)	0	0	0.588235	0.294118	0.117647

#### 2.2.5 Fuzzy comprehensive evaluation

Multiplying the index weight vector W of the factor set by the corresponding fuzzy evaluation matrix R can obtain the corresponding evaluation vector A. Multiplying the evaluation vector A by the evaluation language set V yields the fuzzy comprehensive evaluation score, and the calculation formula is P=W·R·V . The total score for the evaluation of the collaborative governance effect of food safety is 3.6363, which falls between “important” and “average.”

#### 2.2.6 Reliability and sensitivity testing

The consistency of the AHP matrix has been verified, and the CR is all less than 0.1. Meanwhile, the average deviation rate of the expert scores of 10 groups was recalculated through random sampling. The fluctuation range of the results was ±3.5%, indicating that the weighting results were stable and reliable. If the questionnaire was scored using the Likert scale, the Cronbach’s *α* value reached 0.875, indicating a relatively high reliability of the questionnaire.

To test the robustness of the model, this study conducted sensitivity tests by increasing and decreasing the key weights (government regulatory capacity and public participation) by 10%, respectively. The results showed that the change in the comprehensive score did not exceed ±0.03, indicating that the AHP-FCE model has strong robustness and stability.

### 2.3 Summary

This chapter constructs an evaluation index system for the collaborative governance of food safety in Jilin Province based on the DPSIR framework, clarifying the weight structure of four secondary indicators and their tertiary indicators. The research determines the weight distribution through expert interviews and the AHP analysis method, and realizes the quantitative evaluation of the governance level by using the fuzzy comprehensive evaluation method. The results show that the overall level of collaborative governance of food safety in Jilin Province is at the “moderately low” stage, and the government’s regulatory capacity and public participation are the key influencing factors. The model has been tested for consistency and sensitivity, and its reliability is good, providing a methodological and data basis for subsequent result analysis and policy optimization.

## 3 Results

### 3.1 Comprehensive evaluation result

Based on the comprehensive calculation of the AHP-FCE model, the comprehensive index score of fairness in the collaborative governance of food safety in Jilin Province was 3.63 points (out of 5), which was within the range of “average” to “good.” However, behind this comprehensive score lies the profound differentiation and inequality within the governance system. Through in-depth analysis of the four dimensions and their 18 third-level indicators, this study clearly reveals the systematic and unfair distribution picture of the benefits of collaborative governance at the three levels of structure, process and result. Data shows that governance resources, participation opportunities and ultimate guarantee levels do not flow in a homogeneous manner, but form a distinct attenuation gradient along multiple axes such as the geographical boundaries between urban and rural areas, the scale gradient of enterprises and the social and economic capital of individuals, leaving some groups and regions in a relatively vulnerable and marginalized position in the fundamental health protection system of food safety. Table 5 presents the fuzzy comprehensive evaluation results and grade distribution of each secondary indicator.

**Table 5:** The comprehensive evaluation results of the collaborative governance performance of food safety in Jilin Province.

Second-level indicators	Weight	Synthesis score	Grade description
A1. Government regulatory capacity (G)	0.3850984	4	The policy enforcement is strong and the degree of law enforcement standardization is relatively high
A2. Implementation of corporate responsibility (E)	0.11727	3	The implementation of the main responsibility is not sufficient
A3. Third parties and inspection quality (T)	0.2438468	3.5	The detection mechanism is well-developed but has insufficient coverage
A4. Public participation (C)	0.2537846	3.5	The enthusiasm of the public for supervision needs to be enhanced

### 3.2 Structural equity deficit: gradient differences in resource allocation

As the structural elements that form the foundation of the entire governance system, their uneven distribution poses a primary and profound challenge to fairness. In the dimension of government regulatory capacity with the highest weight (0.385), the data reveals a distinct “center-periphery” pattern, which means the regulatory resources in Jilin Province are highly concentrated in the central city, while the distribution pattern is relatively weak in the peripheral and rural areas. Specifically, the Gini coefficient for regulatory manpower and funds remains at a high level, indicating that administrative resources are highly concentrated in various cities within the province, especially in the central cities. Further analysis of the urban–rural ratio reveals that the number of regulatory personnel per 10,000 people in rural areas is significantly lower than that in urban areas, creating a geographical gap in regulatory density. Meanwhile, the targeted analysis of supervision and sampling inspection has exposed blind spots in the risk monitoring network: although the overall sampling inspection coverage rate is acceptable, a large number of sampling inspection batches have flowed to large supermarkets and well-known enterprises with standardized management and relatively low risks, while the coverage of urban–rural fringe areas, rural markets and food micro and small business forms where food safety hazards are more concentrated is obviously insufficient. This deviation in resource allocation is intensified in the law enforcement response stage - data shows that the average on-site response time of market supervision departments for complaints and reports in remote towns and townships is about 40% longer than that in urban areas. The delay in procedural justice essentially weakens the right of consumers in disadvantaged areas to obtain timely relief. On the other hand, in the dimension of third-party testing quality (weight 0.244), the inequality in service accessibility is equally prominent. The balance index of the distribution of testing institution networks is relatively low. Laboratories with authoritative qualifications are highly concentrated in major cities such as Changchun, which has led to a shortage of professional testing services in some counties. More importantly, although the transparency of service charges has improved, the survey found that the penetration rate of preferential testing policies for small and micro enterprises is less than 30%, and the high compliance costs have become a substantive barrier for small and micro entities to enhance quality and access modern supply chains. The gradient differences of the above-mentioned structural resources jointly lay the physical and institutional foundation for the insufficient fairness of the governance system.

### 3.3 Process equity imbalance: stratification of participation opportunities and capabilities

The participation opportunities and action capabilities in the governance process are not equally open to all. Socioeconomic status has become a key screening mechanism here, leading to significant imbalances in process fairness. In the dimension of enterprise responsibility implementation (weight 0.117), the data shows a severe phenomenon of scale differentiation. The certification rate of food safety management systems varies greatly between large leading enterprises and small and micro enterprises. The latter’s certification rate is less than 15%, reflecting a huge gap in their ability to manage in a standardized manner. The completeness of traceability information also shows a clear “scale gradient.” The product traceability chains of small and micro enterprises are generally shorter and the rate of missing key information is high. This makes it difficult for them to quickly locate and recall risks after they occur, and also reduces regulatory efficiency. The investigation into the coverage and effectiveness of training for practitioners further confirmed that the proportion of practitioners in small and micro enterprises and mobile vendors who have received systematic and regular training and the pass rate of knowledge tests are significantly lower than those of employees in large and medium-sized enterprises, indicating that there are weak links in the “human defense” link at the forefront of risk prevention and control. In the dimension of public participation (weight 0.254), the stratification of opportunities and effectiveness is more complex. Measurements of food safety risk perception and knowledge levels show that there are systematic differences in public cognition among people with different educational backgrounds and places of residence: highly educated individuals and urban residents tend to make judgments based on scientific information, while those with lower education and rural residents rely more on traditional experience and are more susceptible to the influence of false information. An analysis of the utilization efficiency of complaint and reporting channels reveals the gap in rights protection capabilities under the “digital divide”: young and high-income groups familiar with the Internet can more efficiently use online platforms to protect their rights and obtain feedback, while the older adults and low-income groups are more dependent on traditional channels and generally have a lower evaluation of the convenience of the handling process and the satisfaction with the results. Furthermore, the assessment of policy consultation and risk communication activities has found that the participants in such activities are still mainly industry representatives and experts and scholars, while the substantive participation of ordinary consumers, especially migrant workers and housewives, is low. This has led to some key stakeholders being in a state of “silence” during the policy-making process. These procedural barriers make it difficult for the governance system to absorb diverse voices and effectively empower all relevant parties.

### 3.4 Result-related fairness confirms: the unequal distribution of risk, trust and satisfaction

The dual unfairness in structure and process will eventually be transmitted and solidified into systematic differences at the outcome level, directly affecting the health risk exposure levels and social psychological feelings of different groups. The comprehensive assessment results of this study provide strong evidence for this. Although no direct causal modeling was conducted, spatial correlation analysis indicates that regions with weak regulatory resources and low accessibility to testing services have significant spatial overlaps with those with relatively high historical sampling inspection failure rates. This strongly suggests that there may be geographical inequalities in the actual exposure to food safety risks, and residents in resource-scarce areas may face higher actual risks. More directly, there is a stratified comparison of public trust. The data shows that the public’s trust in the effectiveness of government regulation and the self-discipline level of enterprises is significantly positively correlated with social and economic indicators such as an individual’s income and educational attainment. In regions where governance resources are insufficiently invested and public services are poorly accessible, the overall level of public trust in the food safety system is significantly low, making it easy to fall into a vicious cycle of “low investment - low trust - low willingness to cooperate,” which weakens the foundation of social co-governance. Most crucially, the comprehensive model reveals a structural contradiction: The high score in the “government regulatory capacity” dimension (4.0 points, weight 0.385) mainly reflects the effectiveness of the administrative system’s own construction. However, this strong leading force has not been effectively transformed into a collaborative lever for enhancing “corporate responsibility implementation” (3.0 points, weight 0.117) and bridging the “public participation gap.” This indicates that the current governance model, which is centered on the government and emphasizes administrative efficiency, has institutional shortcomings in mobilizing and integrating market and social forces and ensuring their fair participation. The “strong government dominance” feature of the governance system has, to some extent, failed to effectively counter the spontaneous forces of fairness imbalance in the market and social fields caused by differences in capital and capacity, ultimately resulting in the failure to fully realize the expected benefits of collaborative governance - that is, fairness benefiting all.

## 4 Discussion

To effectively transform food safety governance into a positive force promoting health equity for all, it is necessary to drive a fundamental shift in the policy paradigm from “pursuing scale and efficiency” to “practicing precise fairness.” Based on the analytical framework of the DPSIR model and the empirical findings of this study, we propose the following systematic and operational policy recommendations.

The findings of this study regarding the unfairness in the structure and procedures of food safety governance are in line with the conclusions of relevant international research. For instance, studies that apply multi-criteria decision-making methods to evaluate food safety and security in other contexts also emphasize the crucial role of fair resource allocation and inclusive participation. Ocampos applied the fuzzy AHP-TOPSIS technology to identify sustainable manufacturing strategies for food production, emphasizing that considerations of fairness and justice are indispensable parts of building a resilient food system, rather than merely technical efficiency (21). Similarly, research on prioritizing climate change and food security, applying AHP and social network Analysis (SNA) also indicates that the effectiveness of governance is often hindered by uneven influence among stakeholders and unequal access to resources (22), which is consistent with the characteristics of “strong government, weak society, and weak enterprises” that we have observed. The experience of applying the AHP-FCE framework in environmental and food safety evaluations in other regions [such as urban water cycle health assessment (14)] also confirms that such integrated models are effective in capturing the performance of complex multi-dimensional systems, including the equity dimension. These cross-regional similarities enhance the validity of our research results and indicate that the equity deficit discovered in Jilin Province is not an isolated case, but rather reflects the common challenges faced in the process of transforming from top-down regulation to inclusive and multi-subject governance under different governance contexts.

The multi-dimensional evaluation framework centered on fairness developed in this study stands in sharp contrast to most previous governance performance research that focused on overall compliance rate, regulatory coverage rate or emergency response speed. Traditional assessment often regards “efficiency” and “fairness” as potentially conflicting goals, or marginalizes the fairness dimension in the assessment. The innovation of this framework lies in systematically integrating “structural equity,” “process equity” and “outcome equity” into an operational evaluation index system. It not only responds to the question of “whether governance is effective,” but also delves deeper into “for whom governance is effective and where it is effective,” thereby providing new analytical tools and empirical evidence for understanding and solving the problem of uneven distribution of governance outcomes.

At the “Driving Force” level, the primary task is to carry out value reshaping and top-level design. Local governments should establish “minimizing the gap in food safety guarantees among different groups and regions” as a core strategic goal that is on par with “maintaining a high overall pass rate.” It is suggested that the “Action Plan for Promoting Food Safety Fairness in Jilin Province” be formulated and released, clearly incorporating fairness indicators (such as resource balance index, participation inclusiveness index, satisfaction group difference coefficient, etc.) into the annual performance assessment system for governments at all levels and relevant departments. Through institutionalized accountability and incentives, the abstract concept of fairness should be transformed into specific governance action guidelines.

At the “Pressure” and “State” levels, policy intervention needs to be more targeted and dedicated to addressing the identified deficiencies in fairness. In response to the structural imbalance of resources, a dynamic fiscal transfer payment mechanism should be established based on indicators such as the Gini coefficient of regulatory resources. A “Special Fund for Enhancing the Governance Capacity of Food Safety in Grassroots and Rural Areas” should be set up, specifically for supplementing regulatory personnel in remote areas, and providing modern rapid testing equipment and mobile laboratories. In response to the differences in the capabilities of market entities, it is necessary to design precise enabling tools. For instance, “Food Safety Compliance Service Vouchers” should be issued to small and micro enterprises to subsidize their certification consultation and product testing fees. At the same time, the fulfillment of “fair responsibilities” by enterprises (such as ensuring employee training, providing capacity support to vulnerable suppliers, and maintaining information transparency) should be included in the enterprise credit evaluation, and preferential policies in financing, procurement and other aspects should be granted. In response to the barriers to social participation opportunities, it is necessary to develop an “inclusive participation toolkit,” including designing voice navigation complaint systems for the older adults and people with disabilities, setting up physical food safety information stations in communities and rural areas, and ensuring through legislation or administrative regulations that in the process of food safety standard formulation, policy hearings and other links, Consumer representatives, especially those of disadvantaged groups, occupy the minimum proportion stipulated by law, and their right to speak is guaranteed procedurally.

At the “Impact” and “Response” levels, it is urgently necessary to build a sensitive and intelligent fairness monitoring and response system. Firstly, multi-source data should be integrated to develop an “Intelligent Monitoring Dashboard for Food Safety Fairness,” achieving real-time visualization and early warning of key fairness indicators such as the distribution of resources in each county and city, the targeting of random inspections, the timeliness of complaint response, and public trust. This enables decision-makers to have an intuitive understanding of the geographical and group distribution of fairness shortcomings, just as they would pay attention to the pass rate of random inspections. Secondly, a new model of “fair regulation based on risk assessment” should be implemented, and more regulatory resources should be proactively allocated to the high-risk weak links revealed by the monitoring dashboard - that is, specific areas (such as urban–rural fringe areas) and specific business types (such as small and micro catering, food vendors around schools) where fairness indicators remain persistently low but risk hazards are relatively high. Finally, it is necessary to improve the mechanism of “fair-oriented emergency management and risk communication.” When a food safety incident occurs, the emergency response plan should give priority to assessing the potential impact of the incident on vulnerable groups such as children, the older adults, and low-income communities, and ensure that rescue and compensation measures can cover them fairly and promptly. All risk communication materials must be produced in multiple versions (such as short text versions, illustrated versions, and dialect audio versions) to ensure that the public of different educational levels and information acquisition habits can accurately and indiscriminately understand the nature of the risks and the necessary protective actions.

In conclusion, this study ultimately calls for a fundamental ideological shift: The ultimate mission of food safety governance should not merely be to pursue the bottom-line safety of “no incidents” or the technical performance of “high pass rates”, but should be dedicated to achieving the goal that “no one is forced to bear disproportionate food safety risks due to their socioeconomic status, residential area or identity characteristics.” Only through the reengineering of the governance system with fairness and justice as its core values, and precisely targeting the groups and regions that need resources, opportunities and capacity building, can a solid social foundation for the realization of the “Healthy China” strategy be truly consolidated, making food safety a powerful booster for promoting the health and fairness of all citizens, rather than a new line of social differentiation.

## 5 Limitation

This research has certain limitations in design and implementation. Firstly, the research conclusions are mainly based on the survey data of Jilin Province in 2025, with limited geographical coverage and a relatively small sample size (*n* = 33). Moreover, they are mainly based on expert scores and cross-sectional data. Therefore, the conclusions have deficiencies in terms of universality in other regions across the country, long-term dynamic evolution trends, and strict causal inference capabilities. Secondly, although the research method adopts the AHP-FCE model and fuzzy processing to reduce subjectivity, the setting of indicator weights, expert judgments, and the quantification of some soft indicators (such as public trust and participation depth) still cannot completely avoid subjective biases. Moreover, the systematic policy recommendations proposed have not yet been tested in practice, and their practical operability, cost and effectiveness need to be further verified.

## 6 Suggestions for future research

Based on the findings and limitations of this study, the following suggestions are put forward for future research.

Future research can be expanded to the whole country or multiple typical provinces for comparative analysis to test the universality and regional differences of the issue of fairness in food collaborative governance. It is suggested to conduct long-term tracking studies or panel data surveys to capture the dynamic evolution trends of governance equity and the long-term effects of policy interventions. In terms of methods, big data, spatial analysis and causal inference models (such as the difference-in-differences method) can be further integrated to strengthen the empirical test of the fair transmission mechanism of “structure - process - result.” In addition, it is necessary to enhance interdisciplinary cooperation, introduce perspectives from public health, sociology, etc., deepen the understanding of the intrinsic connection between health equity and governance equity, and explore the development of more systematic and operational equity monitoring indicators and assessment tools to promote the transformation of food safety governance towards a “precise equity” paradigm.

## 7 Conclusion

Based on the DPSIR model and the comprehensive evaluation results of AHP-FCE, this study systematically analyzed the operational logic and practical performance of the collaborative governance system for food safety in Jilin Province. The DPSIR framework clearly Outlines the causal chain of this system, as shown in Figure 1: social and economic development and consumption upgrade, as the fundamental driving forces, have promoted the growth of governance demands; However, the complexity of the industrial structure, the insufficiency of enterprises in implementing their main responsibilities, and the imbalance in information construction jointly constitute the internal pressure faced by governance. Against this backdrop, the current governance situation, defined by the government regulatory system, enterprise quality control and the level of social participation, directly determines the direction of core impacts such as food safety incident risks, public trust levels and the sustainable development of the industry. The linkage mechanism among the government, enterprises and society, the digital regulatory capacity and the completeness of the risk prevention and control system all centrally reflect the system’s response efficiency to challenges.

Through the quantitative calculation of the AHP-FCE model and the qualitative analysis of expert interviews, the comprehensive score of the collaborative governance of food safety in Jilin Province was 3.63 points (out of 5 points), which was generally at a “good” level. This result indicates that the governance system of this province has laid a foundation and achieved initial success in terms of institutional construction and policy implementation. Specifically, the government’s regulatory capacity scored the highest (4.0 points), highlighting the significant advantages of the Jilin Provincial Government in terms of a complete regulatory system, standardized law enforcement, and cross-departmental coordination, and demonstrating strong institutional enforcement and policy synergy. The quality of third-party testing and public participation both received 3.5 points, indicating that social forces and professional institutions have played an auxiliary role in governance. However, there is still significant room for improvement in the depth of information sharing and the efficiency of data integration. In contrast, the score for corporate responsibility fulfillment is only 3.0 points, making it the weakest link in the system. This reflects that the main body of enterprises is still passive in terms of responsibility awareness and self-discipline mechanisms, and lacks internal motivation.

From the perspective of the overall evolution trend, the food safety governance system in Jilin Province is undergoing a structural transformation from “single-center supervision” to “multi-subject collaborative governance.” The construction of institutionalization and informatization has achieved phased results, marking a crucial transitional period for the system to move from “institutional establishment” to “mechanism maturity.” However, this transformation process still faces multiple structural challenges: issues such as blurred boundaries of responsibilities, fragmented collaboration mechanisms, and poor intercommunication among information systems have not been fundamentally resolved. The participation of enterprises and social entities remains at the surface level and fails to form a deep synergy, which leads the government to continuously bear an excessive regulatory burden and further restricts the improvement of the overall efficiency and sustainability of the governance system.

Further focusing on the perspective of fairness, this study finds that there exists a multi-dimensional and systematic “fairness deficit” in the collaborative governance of food safety in Jilin Province. This deficit is no accident but is deeply rooted in the structure and process of the governance system. Specifically, it is manifested in the structural mismatch of governance resources in terms of space and hierarchy, making rural and underdeveloped areas become low-lying areas for supervision and services. Market entities and the general public face significant barriers to opportunities and capabilities during the participation process, resulting in small and micro enterprises and groups with low socioeconomic status being at a disadvantage in terms of responsibility fulfillment and interest expression. However, the cross-departmental and cross-subject collaborative mechanisms remain loose and formalistic, making it difficult to pool a systematic synergy to correct unfairness. These factors are intertwined, preventing food safety, a fundamental public good, from covering all groups fairly and objectively exacerbating health risk inequalities based on region, occupation and class.

In-depth analysis reveals that the fair transmission chain of “structure - process - result” has broken at a key link. Although the government has shown strong performance in “structural” investment, this advantage of administrative dominance has not been effectively transformed into extensive, fair and in-depth “process-oriented” participation. The intrinsic motivation for responsibility among enterprises, especially micro and small entities, remains insufficient. Public participation is clearly stratified in terms of capabilities and opportunities, ultimately making it difficult to achieve “result-oriented” fairness - that is, the equalization of risk exposure, safety perception and trust levels. A prominent contradiction lies in the fact that the high-weight government regulatory efficiency has not effectively driven the low-weight corporate responsibility fulfillment and the quality of public participation, revealing that in the current collaborative model, administrative pressure has not fully stimulated fair actions at the market and social levels, and the value of fairness has been significantly diluted in the process of transmission.

These findings collectively point to a core governance revelation: while pursuing management efficiency, the dimension of fairness must be systematically and measurably incorporated into governance goals and evaluation systems. Fairness should not merely be an abstract value that is called for, but must become a concrete governance dimension that is continuously monitored, evaluated and held accountable. This means that future food safety governance policies need to set clear fairness indicators (such as the resource balance index and the participation inclusiveness index) just as they set the “sampling inspection pass rate” target, and establish corresponding data collection and monitoring feedback mechanisms.

Therefore, this study ultimately calls for a fundamental paradigm shift: The ultimate mission of food safety governance should be dedicated to ensuring that no one is forced to bear disproportionate food safety risks due to their socioeconomic status, residential area, or identity characteristics. Only through the reconstruction of a governance system based on evidence and oriented towards fairness, accurately identifying and supporting the most vulnerable groups and regions, can the social foundation of the “Healthy China” strategy be truly consolidated, and food safety governance become a powerful engine for promoting health equity for all.

## Data Availability

The original contributions presented in the study are included in the article/Supplementary material, further inquiries can be directed to the corresponding author.
