# Supplementary material for: Fairness evaluation of collaborative governance of food safety in Jilin Province based on AHP-FCE model: multi-dimensional difference analysis and policy implications research
Source: Front Public Health. 2026 Mar 3;14:1770663. doi: 10.3389/fpubh.2026.1770663 (PMC12992232; doi:10.3389/fpubh.2026.1770663)
Supplement: Supplementary file 1 [file Data_Sheet_1.docx]

Supplementary:

Supplementary table 1: Evaluation results of the collaborative governance effect on food safety of pre-prepared meals.

| **First-level indicator** | **Second-level indicators** | **Third-level indicators** | **Very Unimportant** | **Unimportant** | **Generally** | **Important** | **Very Important** |
| --- | --- | --- | --- | --- | --- | --- | --- |
|  | A1. Government regulatory capacity（G） | Supervise the Gini coefficient/urban-rural ratio of human resources and funds G1) | 0 | 0 | 5 | 11 | 1 |
|  |  | The coverage and targeting of food safety supervision and sampling inspection (G2) | 0 | 1 | 8 | 6 | 2 |
|  |  | The timeliness of responses to illegal acts and regional differences (G3) | 0 | 2 | 6 | 8 | 1 |
|  |  | The frequency and case type distribution of cross-departmental collaborative law enforcement (G4) | 0 | 2 | 4 | 11 | 0 |
|  |  | The transparency and understandability of regulatory information disclosure (G5) | 0 | 0 | 10 | 5 | 2 |
|  |  | The certification rates of food safety management systems in enterprises of different scales (E1) | 0 | 1 | 2 | 9 | 5 |
| A. The effect of collaborative governance on food safety | A2. Implementation of corporate responsibility（E） | The enterprise scale gradient of traceability information completeness (E2) | 0 | 1 | 3 | 12 | 1 |
|  |  | The differences in the coverage and effectiveness of training for practitioners (E3) | 0 | 1 | 8 | 8 | 0 |
|  |  | The authenticity of the self-inspection report submission rate and the problem discovery rate (E4) | 0 | 1 | 4 | 9 | 3 |
|  |  | Industry and scale differences in the proportion of food safety investment to revenue (E5) | 0 | 1 | 4 | 10 | 2 |
|  |  | The regional equilibrium index of the distribution of testing institution outlets (T1) | 0 | 1 | 4 | 8 | 4 |
|  | A3. Third parties and inspection quality（T） | The transparency of service charge standards and the affordability of small and micro enterprises (T2) | 0 | 0 | 6 | 7 | 4 |
|  |  | The stability of the timeliness of issuing test reports and the fulfillment rate of commitments(T3) | 0 | 0 | 3 | 11 | 3 |
|  |  | The openness of proficiency testing and flight inspection results and the differences among institutions (T4) | 0 | 3 | 7 | 5 | 2 |
|  |  | Group differences in food safety risk perception and knowledge level (C1) | 0 | 3 | 7 | 4 | 3 |
|  | A4. Public participation（C） | Analysis of the Differences in the Utilization Efficiency of Complaint and Reporting Channels (C2) | 0 | 2 | 5 | 9 | 1 |
|  |  | The representativeness and accessibility of participation in policy consultation and risk communication activities (C3) | 0 | 0 | 6 | 10 | 1 |
|  |  | The deviation in the attention paid to food safety issues by the media and social platforms (C4) | 0 | 3 | 5 | 8 | 1 |

Supplementary table 2: Fuzzy membership degree matrix.

| **First-level indicator** | **Second-level indicators** | **Third-level indicators** | **Very Unimportant** | **Unimportant** | **Generally** | **Important** | **Very Important** |
| --- | --- | --- | --- | --- | --- | --- | --- |
|  | A1. Government regulatory capacity（G） | Supervise the Gini coefficient/urban-rural ratio of human resources and funds G1) | 0 | 0 | 0.294118 | 0.647059 | 0.058824 |
|  |  | The coverage and targeting of food safety supervision and sampling inspection (G2) | 0 | 0.058824 | 0.470588 | 0.352941 | 0.117647 |
|  |  | The timeliness of responses to illegal acts and regional differences (G3) | 0 | 0.117647 | 0.352941 | 0.470588 | 0.058824 |
|  |  | The frequency and case type distribution of cross-departmental collaborative law enforcement (G4) | 0 | 0.117647 | 0.235294 | 0.647059 | 0 |
|  |  | The transparency and understandability of regulatory information disclosure (G5) | 0 | 0 | 0.588235 | 0.294118 | 0.117647 |
|  |  | The certification rates of food safety management systems in enterprises of different scales (E1) | 0 | 0.058824 | 0.117647 | 0.529412 | 0.294118 |
| A. The effect of collaborative governance on food safety | A2. Implementation of corporate responsibility（E） | The enterprise scale gradient of traceability information completeness (E2) | 0 | 0.058824 | 0.176471 | 0.705882 | 0.058824 |
|  |  | The differences in the coverage and effectiveness of training for practitioners (E3) | 0 | 0.058824 | 0.470588 | 0.470588 | 0 |
|  |  | The authenticity of the self-inspection report submission rate and the problem discovery rate (E4) | 0 | 0.058824 | 0.235294 | 0.529412 | 0.176471 |
|  |  | Industry and scale differences in the proportion of food safety investment to revenue (E5) | 0 | 0.058824 | 0.235294 | 0.588235 | 0.117647 |
|  |  | The regional equilibrium index of the distribution of testing institution outlets (T1) | 0 | 0.058824 | 0.235294 | 0.470588 | 0.235294 |
|  | A3. Third parties and inspection quality（T） | The transparency of service charge standards and the affordability of small and micro enterprises (T2) | 0 | 0 | 0.352941 | 0.411765 | 0.235294 |
|  |  | The stability of the timeliness of issuing test reports and the fulfillment rate of commitments(T3) | 0 | 0 | 0.176471 | 0.647059 | 0.176471 |
|  |  | The openness of proficiency testing and flight inspection results and the differences among institutions (T4) | 0 | 0.176471 | 0.411765 | 0.294118 | 0.117647 |
|  |  | Group differences in food safety risk perception and knowledge level (C1) | 0 | 0.176471 | 0.411765 | 0.235294 | 0.176471 |
|  | A4. Public participation（C） | Analysis of the Differences in the Utilization Efficiency of Complaint and Reporting Channels (C2) | 0 | 0.117647 | 0.294118 | 0.529412 | 0.058824 |
|  |  | The representativeness and accessibility of participation in policy consultation and risk communication activities (C3) | 0 | 0 | 0.352941 | 0.588235 | 0.058824 |
|  |  | The deviation in the attention paid to food safety issues by the media and social platforms (C4) | 0 | 0.176471 | 0.294118 | 0.470588 | 0.058824 |

**AHP Questionnaire on the Importance of Evaluation Indicators for the Synergistic Governance Effect of Pre-prepared Food Safety**

**Questionnaire Instructions**

Dear interviewee:

This questionnaire aims to understand your views on the importance of various evaluation indicators in the collaborative governance of food safety for pre-prepared meals. Please rate the importance of each indicator based on your own understanding and experience. Your opinions will provide an important reference for building a scientific and reasonable evaluation system. The questionnaire is conducted anonymously, and all information is only used for statistical analysis.

Scoring instructions

Please use the Likert 5-point scale to rate the importance of each indicator. The higher the score, the more important you consider the indicator to be. (1= very unimportant, 5= very important)

**Part One: Basic Information**

1. What field do you belong to? (Single choice)
2. Government regulatory agencies
3. Food production enterprises
4. Third-party testing institutions
5. Consumer organization
6. The number of years you have been engaged in or focused on in your field is_____

**Part Two: Scoring of the Importance of First-level Indicators**

Please evaluate the overall significance of the following four dimensions in the collaborative governance of food safety for pre-prepared meals.

| Number | First-level indicator | Importance score (1-5 points) |
| --- | --- | --- |
| 1 | A1. Government regulatory capacity（G） |  |
| 2 | A2. Implementation of corporate responsibility（E） |  |
| 3 | A3. Third parties and inspection quality（T） |  |
| 4 | A4. Public participation（C） |  |

**Part Three: Scoring of the Importance of Secondary Indicators**

Please evaluate the relative importance of the following specific indicators within their respective dimensions.

A1. Government regulatory capacity（G）

| Number | Second-level indicator | Importance score (1-5 points) |
| --- | --- | --- |
| 1 | Supervise the Gini coefficient/urban-rural ratio of human resources and funds G1) |  |
| 2 | The coverage and targeting of food safety supervision and sampling inspection (G2) |  |
| 3 | The timeliness of responses to illegal acts and regional differences (G3) |  |
| 4 | The frequency and case type distribution of cross-departmental collaborative law enforcement (G4) |  |
| 5 | The transparency and understandability of regulatory information disclosure (G5) |  |

A2. Implementation of corporate responsibility（E）

| Number | Second-level indicator | Importance score (1-5 points) |
| --- | --- | --- |
| 1 | The certification rates of food safety management systems in enterprises of different scales (E1) |  |
| 2 | The enterprise scale gradient of traceability information completeness (E2) |  |
| 3 | The differences in the coverage and effectiveness of training for practitioners (E3) |  |
| 4 | The authenticity of the self-inspection report submission rate and the problem discovery rate (E4) |  |
| 5 | Industry and scale differences in the proportion of food safety investment to revenue (E5) |  |

A3. Third parties and inspection quality（T）

| Number | Second-level indicator | Importance score (1-5 points) |
| --- | --- | --- |
| 1 | The regional equilibrium index of the distribution of testing institution outlets (T1) |  |
| 2 | The transparency of service charge standards and the affordability of small and micro enterprises (T2) |  |
| 3 | The stability of the timeliness of issuing test reports and the fulfillment rate of commitments(T3) |  |
| 4 | The openness of proficiency testing and flight inspection results and the differences among institutions (T4) |  |

A4. Public participation（C）

| Number | Second-level indicator | Importance score (1-5 points) |
| --- | --- | --- |
| 1 | Group differences in food safety risk perception and knowledge level (C1) |  |
| 2 | Analysis of the Differences in the Utilization Efficiency of Complaint and Reporting Channels (C2) |  |
| 3 | The representativeness and accessibility of participation in policy consultation and risk communication activities (C3) |  |
| 4 | The deviation in the attention paid to food safety issues by the media and social platforms (C4) |  |

The questionnaire is over. Thank you for your participation and support!
